# HMGB1 concentration measurements in trauma patients: assessment of pre-analytical conditions and sample material

**DOI:** 10.1186/s10020-019-0131-0

**Published:** 2019-12-31

**Authors:** William Ottestad, Ingrid N. Rognes, Erlend Skaga, Cassandra Frisvoll, Guttorm Haraldsen, Torsten Eken, Peter Lundbäck

**Affiliations:** 1 Department of Anaesthesiology, Oslo University Hospital, PO Box 4956 Nydalen, NO-0424 Oslo, Norway; 2 Institute of Clinical Medicine, Faculty of Medicine, University of Oslo, Oslo, Norway; 3 Faculty of Medicine, University of Oslo, Oslo, Norway; 4 K.G. Jebsen Inflammation Research Centre, Institute of Clinical Medicine, Faculty of Medicine, University of Oslo, Oslo, Norway; 5 Department of Pathology, Oslo University Hospital, Oslo, Norway

**Keywords:** HMGB1 protein, Pre-analytical phase, Wounds and injuries, Enzyme-linked immunosorbent assay, Blotting, western

## Abstract

**Background:**

HMGB1 is a mediator of systemic inflammation in sepsis and trauma, and a promising biomarker in many diseases. There is currently no standard operating procedure for pre-analytical handling of HMGB1 samples, despite that pre-analytical conditions account for a substantial part of the overall error rate in laboratory testing. We hypothesized that the considerable variations in reported HMGB1 concentrations and kinetics in trauma patients could be partly explained by differences in pre-analytical conditions and choice of sample material.

**Methods:**

Trauma patients (*n* = 21) admitted to a Norwegian Level I trauma center were prospectively included. Blood was drawn in K_2_EDTA coated tubes and serum tubes. The effects of delayed centrifugation were evaluated in samples stored at room temperature for 15 min, 3, 6, 12, and 24 h respectively. Plasma samples subjected to long-term storage in − 80 °C and to repeated freeze/thaw cycles were compared with previously analyzed samples. HMGB1 concentrations in simultaneously acquired arterial and venous samples were also compared. HMGB1 was assessed by standard ELISA technique, additionally we investigated the suitability of western blot in both serum and plasma samples.

**Results:**

Arterial HMGB1 concentrations were consistently lower than venous concentrations in simultaneously obtained samples (arterial = 0.60 x venous; 95% CI 0.30–0.90). Concentrations in plasma and serum showed a strong linear correlation, however wide limits of agreement. Storage of blood samples at room temperature prior to centrifugation resulted in an exponential increase in plasma concentrations after ≈6 h. HMGB1 concentrations were fairly stable in centrifuged plasma samples subjected to long-term storage and freeze/thaw cycles. We were not able to detect HMGB1 in either serum or plasma from our trauma patients using western blotting.

**Conclusions:**

Arterial and venous HMGB1 concentrations cannot be directly compared, and concentration values in plasma and serum must be compared with caution due to wide limits of agreement. Although HMGB1 levels in clinical samples from trauma patients are fairly stable, strict adherence to a pre-analytical protocol is advisable in order to protect sample integrity. Surprisingly, we were unable to detect HMGB1 utilizing standard western blot analysis.

## Background

High-mobility group box 1 protein (HMGB1) is an evolutionary conserved DNA-binding protein present in all nucleated eukaryotic cells and in platelets. Under physiological conditions, HMGB1 is located predominantly in the nucleus where it acts as a nucleosomal stabilizer and a transcriptional regulator. (Bianchi and Agresti 2005) Outside the cell, HMGB1 acquires a new identity to serve as a powerful mediator of inflammation. (Yang et al. 2013) It is passively released or actively secreted in a multitude of pathological conditions including sepsis, trauma, cancer, and auto-immune diseases, and in many cases a predictable correlation to disease activity is evident. (Harris et al. 2012; Ottestad et al. 2019) The biological actions of HMGB1 are remarkable in their diversity, caused by a marked propensity for post-translational modifications. (Yang et al. 2012)

Several studies have explored HMGB1 concentrations in trauma patients utilizing standard ELISA techniques, however there is a considerable variation in reported concentrations and kinetics even in comparable patient populations. (Ottestad et al. 2019; Peltz et al. 2009; Cohen et al. 2009; Yang et al. 2006; Namas et al. 2016) Although pre-analytical conditions have been reported to account for 46 to 68% of the overall error rate in laboratory testing in general, (Carraro et al. 2012; Plebani 2012) there are no recommended operating procedures for clinical HMGB1 analysis that are widely applied. (Moore et al. 2011) We have therefore explored some crucial aspects for measurement of HMGB1 concentrations in trauma patients.

First, analyses of inflammatory molecules are often biased by sample degradation or ex vivo sample activation. This is especially true for constitutively expressed inflammatory markers. (Skogstrand et al. 2008; Flower et al. 2000) Release of HMGB1 from dying blood cells could also potentially affect total levels in blood samples over time. Further complicating the issue, HMGB1 stability in a given sample could conceivably depend on disease type and state, potentially invalidating experience gained from studies in other disease categories. We have therefore evaluated effects on measured HMGB1 concentrations in samples from trauma patients stored at room temperature for variable time periods prior to centrifugation and freezing, and subjected to long-term storage at − 80 °C with several freeze/thaw cycles after centrifugation.

Second, immunoassays are prone to interference by endogenous factors that alter antibody binding or the measurable concentration of the analyte. (Tate and Ward 2004) Cross-reactivity, unsuspected protein-protein bindings, and lipemia are all known to cause interference, and these factors can depend on type of sample material. (Tate and Ward 2004; Clerico et al. 2018) In line with this, both serum and plasma components that interfere with HMGB1 detection by ELISA systems have been reported, yielding different results in serum vs. plasma depending on disease state. (Urbonaviciute et al. 2007; Basso et al. 2017; Lehner et al. 2012) We have therefore compared HMGB1 levels measured by ELISA in simultaneously acquired serum and plasma samples from patients early after trauma. The suitability of western blot (WB) analysis as an alternative to ELISA was also addressed, as western blotting is less prone to interference by protein-protein interactions due to a denaturing step.

Third, previous studies of HMGB1 in trauma patients have either utilized venous samples only, (Cohen et al. 2009) mainly arterial samples, (Ottestad et al. 2019) an unspecified mixture of arterial and venous samples, (Namas et al. 2016) or not reported the source of their material. (Peltz et al. 2009; Yang et al. 2006) It could be argued that arterial samples may be preferable in order to obtain blood that is not draining from any particular injured body part, (Ottestad et al. 2019) however we are not aware of any study comparing HMGB1 concentration in arterial vs. venous blood after trauma. We have therefore also investigated HMGB1 concentrations in plasma derived from simultaneously acquired arterial and venous samples in trauma patients.

## Methods

Recruitment of trauma patients was performed January 2011 – November 2018 at Oslo University Hospital Ullevål, a Norwegian Level I trauma center. (Ottestad et al. 2019) Patients were recruited by convenience. All patients ≥18 years who met criteria for trauma team activation were eligible for enrollment. Patients with burn injuries and pregnant women were excluded.

Arterial or venous blood was drawn in K_2_EDTA coated tubes and serum tubes (Vacuette 454209 and 367977, Greiner Bio-One International GmbH, Austria) immediately after admission. The tubes were put directly in ice slush after 8–10 inversions, and after 15 min centrifuged at 2500 g at 4 °C for 15 min. Aliquots of the supernatants were immediately transferred to sterile polypropylene tubes (NUNC CryoTubes 479–6843; VWR International AS, Oslo, Norway) and stored at − 80 °C.

In a recent study of HMGB1 kinetics in trauma patients (Ottestad et al. 2019) we have reported HMGB1 plasma concentrations as a function of time after injury from 136 of the patients. In the current study, we have compared previously reported plasma concentration measurements from several time points in 5 of these patients to reanalyzed plasma and to serum samples that were not originally analyzed, but subjected to long-term storage at − 80 °C.

Simultaneous arterial and venous blood samples were acquired in 6 additional patients, four of them both at admission and at approximately 4 h after injury. The latter time point was selected to embrace the previously described “second wave” of HMGB1 release after trauma. (Ottestad et al. 2019) In another 10 patients, admission arterial samples were drawn in five 2 ml EDTA tubes which were stored at room temperature (≈25 °C) for 15 min, 3, 6, 12, and 24 h respectively before centrifugation and freezing. An overview of patients and samples in each group is displayed in Table 1.

**Table 1:** Characteristics of the three study populations

Characteristics	Sample Storage patients (*n* = 10)	Serum vs. Plasma patients (*n* = 5)	Arterial vs. Venous patients (*n* = 6)
Demographics			
Sex (male: female)	7: 3	3: 2	6: 0
Age (years)	48 (29–68; 19–78); 10	54 (30–55; 23–55); 5	35 (19–52; 17–57); 6
Pre-injury ASA PS (ASA I: II: III)	5: 3: 2	3: 1: 1	5: 0: 1
Injuries			
Mechanism of injury (blunt: penetrating)	10: 0	4: 1	5: 1
NISS	34 (22–43; 12–75); 10	41 (22–53; 9–57); 5	41 (7–59; 1–66); 6
ISS	28 (20–32; 5–48); 10	24 (15–46; 9–57); 5	36 (4–49; 1–50); 6
Admission BE (mmol/L)	−4.1 (− 7.3 – − 1.0; − 8.3 – 0.1); 10	−3.4 (− 5.6 – − 2.0; − 6.2 – − 1.6); 4	−2.3 (− 6.2–0.3; − 8.7 – 3.7); 6
HMGB1 analyses			
Admission HMGB1 (ng/mL)^a^	6.57 (3.01–29.9; 1.68–155); 10	50.8 (1.88–91.2; 1.54–97.9); 5	13.2 (8.62–17.7; 7.31–20.4); 6
Time from injury to first sample (hours)^b^	3:07 (1:03–5:44; 0:39–7:26); 9	0:45 (0:31–2:41; 0:29–4:06); 5	1:41 (0:49–2:49; 0:17–3:50); 6
Hospital treatment			
ICU length of stay (days)	6 (2–11; 1–17); 10	7 (3–9; 2–10); 5	6 (2–11; 2–12); 6
Ventilator treatment (y: n)	5: 5	2: 3	5: 1
Outcome			
Dead at 30 days (y: n)	1: 9	1: 4	1: 5
Ventilator-free days	29 (11–30; 0–30); 10	30 (0–30; 0–30); 5	23 (17–29; 0–30); 6

Numbers are given as median (quartiles; range) and number of patients if not otherwise specified. *NISS* New Injury Severity Score, *ISS* Injury Severity Score, *BE* Base Excess, *ICU* intensive care unit, Ventilator-free days, days alive and off ventilator during the first 30 days after trauma. ^a^For “Arterial vs. Venous” group, as median concentration from first arterial sample. ^b^Time from injury missing for one patient in “Sample Storage” group

HMGB1 concentrations were obtained by standard ELISA technique according to manufacturer instruction (HMGB1 ELISA Kit II; ST51011; Shino-Test Corporation, Tokyo, Japan). Serum samples and plasma samples subjected to freeze/thaw and long-term storage were analyzed with a single ELISA kit, however compared with original plasma analyses performed with ELISA kits from a different production batch for our previous study. Analyses of simultaneously acquired arterial and venous samples were done within a single ELISA kit in order to rule out inter-assay variability, in quadruplicate with results reported as median concentrations.

Western blotting was performed with 1 μL plasma or serum diluted in 9 μL H_2_O + 3 μL Laemmli sample buffer (100 mM Tris-HCl pH 6.8, 45% glycerol, 5% sodium dodecyl sulfate, 12.5% 2-mercaptoethanol, and 0.25% bromophenol blue), and incubated at 97 °C for 10 min. Recombinant human HMGB1 (HMGBiotech HM100, Milano, Italy) was used as a positive control in western blot analyses at different spike-in concentrations in normal venous plasma and serum from one healthy subject. SDS-PAGE electrophoresis was performed at 200 V for 30 min using mini-protean TGX gels 4–20% (Bio-Rad Laboratories AB, Oslo, Norway). The gel was transferred to nitrocellulose membrane (Trans-Blot® Turbo™ Mini Nitrocellulose 0.2 μm, BioRad Laboratories AB, Oslo, Norway). Membranes were blocked with 5% milk powder in Tris-Buffered Saline and Tween20 TBS-T for 45 min at room temperature, and rinsed once in TBS-T. The membranes were incubated with primary anti-HMGB1 antibodies (Abcam 18256, Cambridge, UK, or m2G7, kind gift from Professor Helena Erlandsson Harris) (Lundbäck et al. 2016) diluted to 1 μg/mL in 1% milk/TBS-T over night at 4 °C. Gels were then washed 4 × 5 min in TBS-T and incubated with HRP-conjugated anti-rabbit antibody (Jackson ImmunoResearch 711-035-152, Immunolab, Oslo, Norway) or HRP-conjugated anti-mouse antibody (Jackson ImmunoResearch 711-035-151, Immunolab, Oslo, Norway) for 2 h at room temperature, diluted 1:20,000 in 1% milk/TBS-T, followed by extensive washing with TBS-T.

Data analysis was undertaken using JMP 11.2.1 and 13.2.0 (SAS Institute, Cary, NC). A two-tailed *p* ≤ 0.05 was chosen to represent statistical significance. Group comparisons were performed with the Wilcoxon rank-sum test, or with the Wilcoxon signed-rank test for paired measurements. Agreement between methods was assessed with the Bland–Altman method for repeated measures. (Myles 2007; Bland and Altman 2007) Linear mixed model analysis with patient as random effect was utilised to assess correlation between original plasma, serum, and reanalyzed plasma, relations between arterial and venous HMGB1 concentration in simultaneous samples, and HMGB1 concentration change during storage. The reported coefficient of multiple determination (R^2^) estimates the proportion of variation in the response that can be attributed to the model rather than to random error.

## Results

Comparisons between HMGB1 concentrations in samples analyzed early, subjected to long-term storage or stored for a variable time in room temperature before centrifugation and freezing, in plasma vs. serum, and in arterial vs. venous blood were done in three separate groups of trauma patients. Characteristics of the study populations are shown in Table 1.

**Fig. 1:**
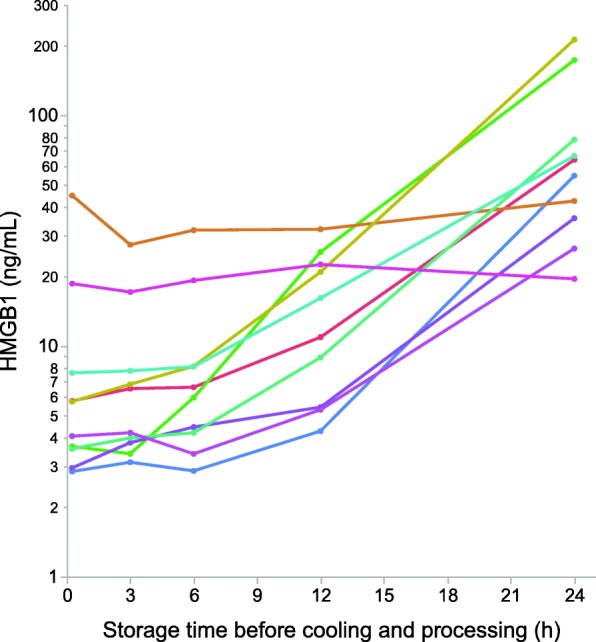
Semilogarithmic plot of HMGB1 concentrations as a function of storage time in room temperature before centrifugation and freezing. Individual patients are color coded

Effects of storage in room temperature before centrifugation and freezing were evaluated in admission samples from 10 trauma patients (Table 1; Fig. 1). There was no significant difference between HMGB1 concentrations after 15 min whether the samples were cooled in ice slush or left in room temperature (*p* = 0.16, Wilcoxon signed-rank test)*.* Concentrations were stable for approximately 3–6 h, but thereafter increased exponentially in samples from eight of the ten patients (Fig. 1). Surprisingly, HMGB1 concentrations from the two patients with highest initial value did not change during the 24 h storage period (linear mixed model; *p* = 0.60).

**Fig. 2:**
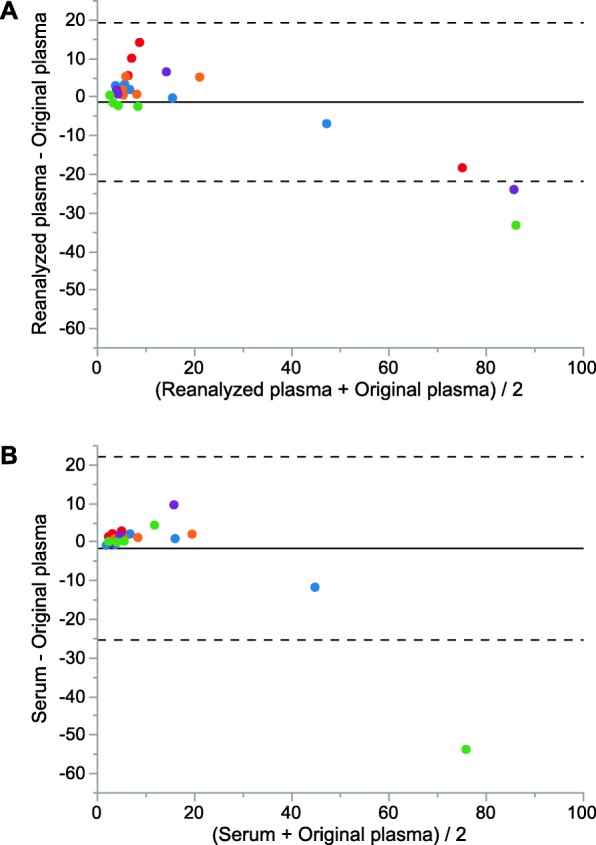
Bland–Altman scatter plot of difference between previously analyzed plasma and reanalyzed plasma (**a**) and previously analyzed plasma and serum (**b**) against the average of the two measurements. Samples from individual patients are color coded

Effects of long-term storage and freeze/thaw cycles on HMGB1 plasma concentration were assessed through reanalysis of 24 plasma samples from five trauma patients (Table 1). Following the initial ELISA-based HMGB1 analyses, each sample was subjected to > 2 years of − 80 °C storage and 3 freeze/thaw cycles. HMGB1 concentrations in original and reanalyzed plasma showed a strong linear correlation (*R*^2^ = 0.986, *p* < 0.0001), however wide limits of agreement (Fig. 2a; mean difference − 1.2 ng/mL, Limits of Agreement [LoA] − 21.7 to 19.3).

Correspondence between HMGB1 serum concentrations and previously analyzed plasma determined by ELISA analysis was assessed in 22 sample pairs from the same patients (Table 1). HMGB1 concentrations in plasma and serum showed a strong linear correlation (*R*^2^ = 0.83, *p* < 0.0001), however wide limits of agreement (Fig. 2b; mean difference − 1.7 ng/mL, LoA − 25.5 to 22.1 ng/mL).

Western blot analyses were performed on simultaneously obtained serum and plasma admission samples from two patients, with ELISA plasma HMGB1 concentrations of 50.8 and 97.9 ng/mL respectively. These are among the highest HMGB1 concentrations reported in our previous study (population median at admission 3.74 ng/mL, range 0.31–223 ng/mL, *n* = 135). (Ottestad et al. 2019) We were unable to detect HMGB1 in any of the samples, utilizing either the polyclonal rabbit antibody or the monoclonal mouse antibody (data not shown). To determine the lowest level of detection by western blot, we diluted recombinant HMGB1 in normal serum and plasma, and were only able to detect HMGB1 bands at spike-in concentrations equal to or above 1000 ng/mL.

**Fig. 3:**
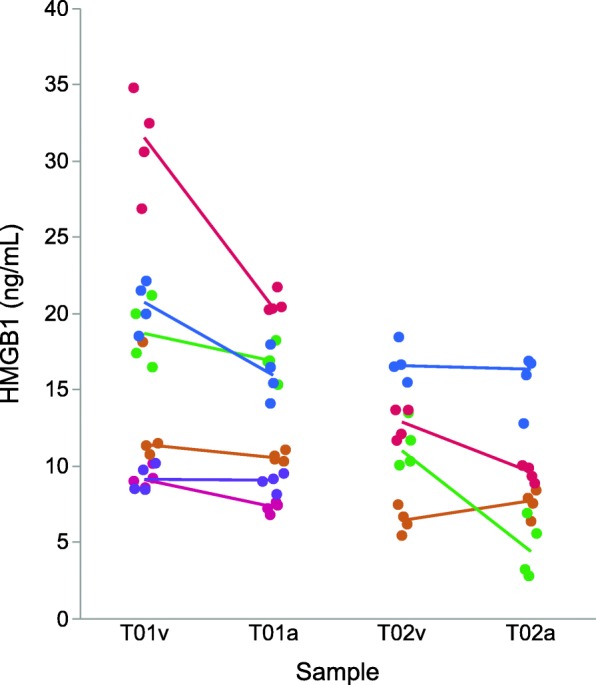
Raw data plot of simultaneously acquired arterial and venous samples. Horizontal axis is marked with venous (v) and arterial (a) samples at T01 (admission) and T02 (approximately 2 h after admission, see text). All samples were analyzed in quadruplicate with results reported as median concentrations. Samples from individual patients are color coded, with a solid line between simultaneously obtained median venous and arterial HMGB1 concentrations

Simultaneously acquired arterial and venous samples were obtained from 6 patients at admission (median 1:41 h after injury, range 0:17–3:50 h, Table 1; T01 in Fig. 3). Samples were also obtained from 4 of the patients at a median of 4:12 h after injury (range 3:56–4:16 h; T02 in Fig. 3). Median absolute time difference between simultaneously acquired samples was 3 min (range 1–4 min); arterial blood was sampled before venous in 6 of the 10 sample pairs. Arterial concentrations were consistently lower than venous (Fig. 3; *p* = 0.01, Wilcoxon signed-rank test), and there was no significant effect of whether the arterial sample was obtained before or after the venous sample (*p* = 0.83, Wilcoxon rank-sum test). Effects of venous HMGB1 concentration on arterial HMGB1 concentration were assessed in a mixed model with patient as random effect. Arterial HMGB1 concentration was 0.60 × venous HMGB1 concentration (95% CI 0.30–0.90, *R*^2^ = 0.68, *p* = 0.002).

## Discussion

We are not aware of any previously published reports comparing venous and arterial blood in clinical samples with respect to systemic HMGB1 levels. Surprisingly, arterial concentrations were 40% lower than venous concentrations, and it is tempting to speculate whether the pulmonary circulation might be a site for HMGB1 clearance. Alternatively, factors in arterial blood may interfere with HMGB1 ELISA detection. HMGB1 undergoes rapid cysteine redox changes, (Zandarashvili et al. 2013) and since ambient oxygen pressure increases when venous blood reaches the pulmonary circulation, HMGB1 oxidation to disulfide or sulfonyl isoforms could potentially alter protein-protein interactions and consequently detection by ELISA. The fraction of free vs. complexed HMGB1 could conceivably also be affected by redox state, as is the case with the alarmin IL33. (Scott et al. 2015)

HMGB1 analyses are often performed on plasma or serum samples that have been subjected to variable duration of storage and sometimes repeated freeze/thaw cycles. Previous studies in non-trauma patients have reported that HMGB1 concentrations in serum samples stored in room temperature for up to 7 days after centrifugation or subjected to freeze/thaw cycles were remarkably stable. (Lehner et al. 2012; Wang et al. 2015) In accordance with this we found a strong linear correlation between concentrations in originally analyzed plasma and plasma subjected to long-term storage and freeze/thaw cycles, however comparison of absolute concentration values was not straightforward due to wide limits of agreement. Despite HMGB1 being fairly stable during storage in room temperature, we found an exponential increase in HMGB1 levels after 3–6 h when blood samples from trauma patients were stored at room temperature before centrifugation and freezing. Surprisingly, HMGB1 concentrations from the two samples with the highest initial HMGB1 value did not exhibit an exponential increase during storage. Neutrophils and thrombocytes have been reported as major vehicles for HMGB1, both cell types are easily activated and short lived, and consequently potential sources of ex-vivo release of HMGB1 in blood samples. Contribution from other cell types can also not be excluded. Although highly speculative, ex-vivo release of HMGB1 might conceivably be limited upon depletion due to extravasation to injured tissues or clot formation following traumatic coagulopathy. Kinetic analysis of more cell type specific markers may be required to explain this observation.

Based on these findings, we speculate that differences in previously reported HMGB1 concentrations in comparable populations of trauma patients (Ottestad et al. 2019; Cohen et al. 2009; Yang et al. 2006; Namas et al. 2016) might be caused by variability in pre-analytical conditions. Peltz et al. (2009) published the first paper to document elevated HMGB1 plasma concentrations after trauma in humans, however their HMGB1 concentrations were 100-fold higher than HMGB1 levels reported in similar studies with comparable populations of trauma patients. (Ottestad et al. 2019; Cohen et al. 2009; Yang et al. 2006; Namas et al. 2016) Notably, in contrast to the other authors, Peltz et al. utilized samples from EDTA tubes previously drawn for clinical indications and processed within 24 h. Storage temperature and time from sampling to centrifugation were not reported, and their results must therefore be interpreted with caution.

HMGB1 concentrations in parallel plasma and serum samples showed strong linear correlation, but wide limits of agreement. Consequently, we recommend that absolute concentration values from plasma and serum samples should be compared with caution, at least in the context of trauma.

The application of immunoassays in complex matrices such as plasma and serum is challenged by interference, (Tate and Ward 2004; Clerico et al. 2018) also known as “matrix effects”. Polyreactive antibodies or unsuspected protein–protein interactions may interfere with antibody detection, decreasing both sensitivity and specificity of the assay and consequently challenging reliable HMGB1 detection in clinical samples. Previous reports have suggested that factors in serum and plasma interfere with HMGB1 detection in ELISA systems. (Urbonaviciute et al. 2007; Bianchi 2009; Abdulahad et al. 2011) HMGB1 is a “sticky” protein that interacts with a multitude of molecules, including proteins, DNA and lipids. In trauma there is an abundance of intracellular proteins leaked from necrotic cells into the circulating plasma, and proteins binding to HMGB1 could potentially negatively impact HMGB1 detection in the ELISA system. ELISA depends on an antibody capture step in order to decrease the complexity of the sample and increase signal-to-noise ratio. Thus, test sensitivity will be affected by sample refinement and type of capture antibody used.

In western blotting, under reducing and denaturing conditions, interfering proteins or other factors will not stay complexed to HMGB1 unless they are covalently bound. Consequently, western blotting could potentially allow for a more unbiased detection of HMGB1. However, we were unable to detect HMGB1 in either serum or plasma from our trauma patients, using two diverse primary antibodies that recognize surface-exposed epitopes. The mAb has a specificity to A-box residue 53–63, and was generated by immunization with recombinant full-length HMGB1. (Qin et al. 2006) The polyclonal Ab has specificity to residue 151–185, including parts of the B-box and linker and both antibodies have been described to be suitable as primary western blot antibodies. However, antibody epitopes may be inaccessible because of sample-specific post-translational modifications (e.g. glycosylation). HMGB1 bands were only detected at spike-in concentrations of 10–50 times the maximum level measured by ELISA in our trauma patients. High protein content in most clinical samples limits the possible sample-loading amount, as expected we experienced problems with unspecific binding and interference with migration at higher serum or plasma volumes than 1 μL/lane. We avoided the use of immunoprecipitation or other enrichment strategies in order to ensure an unbiased approach.

Our study has several limitations. It was a single-center study, and sample size was small and limited to trauma patients only. Reanalyzed plasma and serum samples were compared with plasma analyzed with ELISA assays from a different production batch, and part of the disagreement between original plasma and reanalyzed plasma or original plasma and serum might be explained by inter-assay imprecision which has been reported to be up to 14% for the HMGB1 immunoassay that was used. (Lehner et al. 2012)

## Conclusions

HMGB1 concentration measurements are sensitive to pre-analytical conditions and choice of sample material. Consequently, standard operating procedures for clinical HMGB1 research should be established so that robust conclusions can be drawn when comparing results from different studies. The observation that arterial HMGB1 concentrations were consistently lower than venous concentrations in simultaneously obtained samples is novel and raises the question whether the pulmonary circulation is a site for HMGB1 clearance. Absolute concentration values in plasma and serum must be compared with caution due to wide limits of agreement. In accordance with earlier studies we confirmed that HMGB1 plasma levels are fairly stable in samples subjected to long-term storage and freeze / thaw cycles, however storage in room temperature before centrifugation resulted in exponential increase in HMGB1 concentrations after 3–6 h. The high correlation between concentration measurements before and after long-term storage allows studies of kinetic profiles within subjects despite wide limits of agreement. Somewhat unexpectedly, standard western blot analysis failed to detect HMGB1 at clinically relevant levels in plasma and serum samples from our trauma patients.

## Data Availability

The datasets used and/or analyzed during the current study are available from the corresponding author on reasonable request.

## Abbreviations

DNA: Deoxyribonucleic acid
EDTA: Ethylenediaminetetraacetate
ELISA: Enzyme-linked immunosorbent assay
HMGB1: High mobility group box 1 protein
HRP: Horseradish peroxidase
